# Supplementary material for: Mutations associated with neuropsychiatric conditions delineate functional brain connectivity dimensions contributing to autism and schizophrenia
Source: Nat Commun. 2020 Oct 19;11:5272. doi: 10.1038/s41467-020-18997-2 (PMC7573583; doi:10.1038/s41467-020-18997-2)
Supplement: Supplementary file 3 — Description of Additional Supplementary Files [file 41467_2020_18997_MOESM3_ESM.pdf]

Description of Additional Supplementary Files

File Name: Supplementary Data 1
Description: CWAS beta estimates, ranking of regions by effect size, region and networks labels.
Effect of the 16p11.2 and 22q11.2 CNVs on 2080 connections. Each value is the Beta estimate (z-scored) of the deletion control contrast. (1.1-1.4)
Effect of the three idiopathic psychiatric conditions on 2080 connections. Each value is the Beta estimate (z-scored) of the deletion control contrast. (1.5-1.7)
Seed regions ranked by the average absolute effect size estimate in each of the 7 CWAS (1.8)
Region name and abbreviation legend (1.9).

File Name: Supplementary Data 2
Description: Similarity of individuals with idiopathic psychiatric disorders with deletion FC-signatures. Differences in individual similarity with 16p11.2 and 22q11.2 deletion FC-signatures in individuals with idiopathic psychiatric conditions and controls (2.1-2.6).

File Name: Supplementary Data 3
Description: Association between similarity with deletion FC-signatures and symptom severity. Correlation of individual similarity with 16p11.2 and 22q11.2 deletion FC-signatures and full-scale intelligence quotient (FSIQ) or autism diagnostic observation schedule (ADOS) in ASD among the 10 seed regions with significant FC similarity between ASD and either deletion.

File Name: Supplementary Data 4
Description: Association between FC-signatures and spatial patterns of gene expression. Partial Least Square Regression (PLSR) association between FC signatures (row means/nodal) and spatial patterns of gene expression of CNV region genes. Percentage variance and P-value using two PLSR Components. (4.1)
PLSR association between FC signatures (regional connectivity) and AHBA gene expression of CNV region genes. Percentage variance and P-value using two PLSR Components. (4.2)
Individual Pearson Correlation between FC signatures (row means/nodal) and spatial patterns of gene expression (AHBA). Pearson Correlation, P-value, FDR-p-value, and Correlation-Centiles per Gene (15633). (4.3)
Individual Pearson Correlation between FC signatures (rowmeans/nodal) and spatial patterns of gene expression (AHBA) for 16p11.2 or 22q11.2 genes. Pearson Correlation, P-value, FDR-pvalue-GenomeWide, Correlation-Centiles-GenomeWide, and FDR-pval-CNVgenes per Gene. (4.4)

.
