# Supplementary material for: Mutations associated with neuropsychiatric conditions delineate functional brain connectivity dimensions contributing to autism and schizophrenia
Source: Nat Commun. 2020 Oct 19;11:5272. doi: 10.1038/s41467-020-18997-2 (PMC7573583; doi:10.1038/s41467-020-18997-2)
Supplement: Supplementary file 8 — Reporting Summary [file 41467_2020_18997_MOESM8_ESM.pdf]

nature research

Corresponding author(s): Sebastien Jacquemont

Last updated by author(s): Sep 5, 2020

Reporting Summary

Nature Research wishes to improve the reproducibility of the work that we publish. This form provides structure for consistency and transparency in reporting. For further information on Nature Research policies, see our Editorial Policies and the Editorial Policy Checklist.

Statistics

For all statistical analyses, confirm that the following items are present in the figure legend, table legend, main text, or Methods section.

n/a	Confirmed	
☐	☒	The exact sample size (n) for each experimental group/condition, given as a discrete number and unit of measurement
☐	☒	A statement on whether measurements were taken from distinct samples or whether the same sample was measured repeatedly
☐	☒	The statistical test(s) used AND whether they are one- or two-sided<br>*Only common tests should be described solely by name; describe more complex techniques in the Methods section.*
☐	☒	A description of all covariates tested
☐	☒	A description of any assumptions or corrections, such as tests of normality and adjustment for multiple comparisons
☐	☒	A full description of the statistical parameters including central tendency (e.g. means) or other basic estimates (e.g. regression coefficient) AND variation (e.g. standard deviation) or associated estimates of uncertainty (e.g. confidence intervals)
☐	☒	For null hypothesis testing, the test statistic (e.g. F, t, r) with confidence intervals, effect sizes, degrees of freedom and P value noted<br>*Give P values as exact values whenever suitable.*
☒	☐	For Bayesian analysis, information on the choice of priors and Markov chain Monte Carlo settings
☒	☐	For hierarchical and complex designs, identification of the appropriate level for tests and full reporting of outcomes
☐	☒	Estimates of effect sizes (e.g. Cohen's d, Pearson's r), indicating how they were calculated

Our web collection on statistics for biologists contains articles on many of the points above.

Software and code

Policy information about availability of computer code

Data collection	No data were collected for this study as all analyses were conducted on data that were already collected as part of several large scale consortium data sets.
Data analysis	The processing scripts and custom analysis software used in this work are available in a publicly accessible GitHub repository with instructions on how to set up a similar computation environment and with examples of key visualizations in the paper: https://github.com/surchs/Neuropsychiatric_CNV_code_supplement<br>We used the following python libraries: scikit-learn (0.20.3), nilearn (0.5.0), nibabel (2.3.3), numpy (1.16.2), statsmodels (0.9.0), pandas (0.24.2), abagen toolbox (version 0.0.5).<br>We used the following R libraries: circlize (version 0.4.10) ,ggplot2 (version 3.2.0), igraph (version 1.2.5), UpSetR (version 1.4.0), ggraph (version 2l.0.3), corrplot (version 0.84).

For manuscripts utilizing custom algorithms or software that are central to the research but not yet described in published literature, software must be made available to editors and reviewers. We strongly encourage code deposition in a community repository (e.g. GitHub). See the Nature Research guidelines for submitting code & software for further information.

Data

Policy information about availability of data

All manuscripts must include a data availability statement. This statement should provide the following information, where applicable:

- Accession codes, unique identifiers, or web links for publicly available datasets
- A list of figures that have associated raw data
- A description of any restrictions on data availability

ABIDE1, COBRE, 16p11.2 SVIP, and 60 % of the schizophrenia data are publicly available.

Around 1/3 of the SZ data (172 out of 484 subjects) can't be shared as participants did not consent to that at the time.
For the 22q11.2 sample, raw data are currently available by request from the PI (CB).
All derived connectome measures used in this study are available through request (SJ).
Beta maps from all Connectome wide association studies (16p11.2 deletion and duplication, 22q11.2 deletion and duplication, ASD, SZ, and ADHD) performed in this study are available in the supplemental table 1 and on GitHub (ttps://github.com/surchs/Neuropsychiatric_CNV_code_supplement). Expression data used in this study are also available in supplemental table 4.

Field-specific reporting

Please select the one below that is the best fit for your research. If you are not sure, read the appropriate sections before making your selection.

☒ Life sciences ☐ Behavioural & social sciences ☐ Ecological, evolutionary & environmental sciences

For a reference copy of the document with all sections, see nature.com/documents/nr-reporting-summary-flat.pdf

Life sciences study design

All studies must disclose on these points even when the disclosure is negative.

Sample size	Previously published studies have shown that 16p11.2 and 22q11.2 deletions have large effect-sizes >0.8 to 1 cohen's d that have been detected with samples of 20 individuals and more (Martin-Brevet et al, 2019; Sun et al, 2018)<br>For autism, schizophrenia and ADHD, we present some of the largest samples available with rsfMRI. Previous studies have shown that schizophrenia and autism have mild effect-sizes on neuroimaging traits (0.3 cohen's d, van Erp et al, 2016; van Rooij et al, 2018, Castellanos, 2016). Therefore, we targeted samples of more than 240 cases and 240 controls to achieve sufficient power. The effect size associated with ADHD is likely lower and remains unclear.<br>References:<br>Martin-Brevet, S. et al. Quantifying the Effects of 16p11.2 Copy Number Variants on Brain Structure: A Multisite Genetic-First Study. Biol. Psychiatry (2018) doi:10.1016/j.biopsych.2018.02.1176.<br>van Erp, T. G. M. et al. Subcortical brain volume abnormalities in 2028 individuals with schizophrenia and 2540 healthy controls via the ENIGMA consortium. Mol. Psychiatry 21, 547–553 (2016).<br>van Rooij, D. et al. Cortical and Subcortical Brain Morphometry Differences Between Patients With Autism Spectrum Disorder and Healthy Individuals Across the Lifespan: Results From the ENIGMA ASD Working Group. Am. J. Psychiatry 175, 359–369 (2018).<br>Sun, D. et al. Large-scale mapping of cortical alterations in 22q11.2 deletion syndrome: Convergence with idiopathic psychosis and effects of deletion size. Mol. Psychiatry (2018) doi:10.1038/s41380-018-0078-5.<br>Castellanos, F. X. & Aoki, Y. Intrinsic Functional Connectivity in Attention-Deficit/Hyperactivity Disorder: A Science in Development. Biol Psychiatry Cogn Neurosci Neuroimaging 1, 253–261 (2016).
Data exclusions	We excluded females from the ABIDE1 dataset due to strong sex imbalance. Preprocessed data were visually controlled for quality of the co-registration, head motion, and related artifacts by one rater. Individuals were excluded from the analysis if co-registration errors could not be fixed. Individuals were also excluded from the analysis if the average framewise displacement after motion censoring exceeded 0.5 mm or if fewer than 40 time frames remained.
Replication	We use all of the resting-state fMRI data available in 16p11.2 and 22q11.2 CNV carriers.<br>Statistical analyses of fMRI connectivity have been technically replicated by 3 authors succesfully, in Python (CM and SU) and R (CS).<br>To our knowledge, there is no independent available CNV dataset for replication.
Randomization	Randomization was not relevant to our study as there were no experimental manipulations
Blinding	Blinding was not relevant to our study as there were no experimental manipulations

Reporting for specific materials, systems and methods

We require information from authors about some types of materials, experimental systems and methods used in many studies. Here, indicate whether each material, system or method listed is relevant to your study. If you are not sure if a list item applies to your research, read the appropriate section before selecting a response.

Materials & experimental systems

n/a	Involved in the study	
☒	☐	Antibodies
☒	☐	Eukaryotic cell lines
☒	☐	Palaeontology and archaeology
☒	☐	Animals and other organisms
☐	☒	Human research participants
☒	☐	Clinical data
☒	☐	Dual use research of concern

Methods

n/a	Involved in the study	
☒	☐	ChIP-seq
☒	☐	Flow cytometry
☐	☒	MRI-based neuroimaging

Human research participants

Policy information about studies involving human research participants

Population characteristics	Models were all adjusted for sex, site, head motion, and age.<br>SVIP data<br>16p11.2 deletion carriers (mean age=12.7, 12/24 males, mean FSIQ=92.5, 4 ASD, 6 ADHD, 0 SZ)<br>16p11.2 duplication carriers (mean age=28.2, 12/23 males, mean FSIQ=94.1, 1 ASD, 1 ADHD, 0 SZ)<br>SVIP non-carriers (mean age=26.7, 46/73 males, mean FSIQ=103.6, 0 ASD, 0 ADHD, 0 SZ)<br><br>UCLA data<br>22q11.2 deletion carriers (mean age=16.8, 20/46 males, mean FSIQ=77.2, 18 ASD, 20 ADHD, 3 SZ)<br>22q11.2 duplication carriers (mean age=16.7, 7/12 males, mean FSIQ=95.7, 3 ASD, 4 ADHD, 0 SZ)<br>UCLA non-carriers (mean age=13, 22/43 males, mean FSIQ=112, 0 ASD, 2 ADHD, 0 SZ)<br><br>ABIDE1 data<br>ASD (mean age=15.9, 225/225 males, mean FSIQ=103.7)<br>non-ASD (mean age=15.7, 234/234 males, mean FSIQ=110.6)<br><br>SZ data<br>SZ (mean age=33.6, 179/241 males)<br>non-SZ (mean age=32.3, 181/242 males)<br><br>ADHD-200 data<br>ADHD (mean age=11.5, 227/289 males, mean FSIQ=106.8)<br>non-ADHD (mean age=12.2, 250/474 males, mean FSIQ=114.2)
Recruitment	Subject from ABIDE were recruited based on the presence of an autism diagnosis, and respective control.<br>Subject from COBRE and Montreal SZ cohorts were recruited based on the presence of a schizophrenia diagnosis and respective control.<br>Subject from ADHD-200 cohort were recruited based on the presence of an ADHD diagnosis and respective control.<br>CNVs cohorts (SVIP and UCLA) were recruited based on the presence of either a 16p11.2 or the 22q11.2 CNVs and respective control.<br>Individuals with the most severe conditions were unlikely to participate due to self-exclusion or inability to perform the scan without moving.
Ethics oversight	All participant recruited in each independent cohort signed a consent form.<br>The current study, which is purely analytical was approved by the IRB (Project 4165) of the Sainte Justine Hospital.

Note that full information on the approval of the study protocol must also be provided in the manuscript.

Magnetic resonance imaging

Experimental design

Design type	Resting-state
Design specifications	This is resting state data without any tasks.
Behavioral performance measures	Full-Scale IQ for all the cohort except for the schizophrenia dataset.<br>ADOS for the ABIDE dataset.<br>PANSS for the schizophrenia dataset.

Acquisition

Imaging type(s)	functional
Field strength	3T
Sequence & imaging parameters	Acquisition parameters were site and dataset specific.
Area of acquisition	Whole-brain scan

Diffusion MRI ☐ Used ☒ Not used

Preprocessing

Preprocessing software	Neuroimaging Analysis Kit (NIAK version 0.12.4)

Normalization	Non-linear
Normalization template	MNI152
Noise and artifact removal	Each data set was corrected for differences in slice acquisition time. Head motion parameters were estimated by spatially re-aligning individual timepoints with the median volume in the time series. This reference median volume was then aligned with the individual anatomical T1 image, which in turn was co-registered onto the MNI152 template space using an initial affine transformation, followed by a nonlinear transformation. Each individual timepoint was mapped to the MNI space using the combined spatial transformations. Slow frequency drifts were modeled on the entire time series as discrete cosine basis functions with a 0.01 Hz high-pass cut-off.
Volume censoring	The first four volumes of each rs-fMRI time series were discarded to allow for magnetization to reach a steady state. Timepoints with excessive in-scanner motion (greater than 0.5 mm framewise displacement) were censored from the time series by removing the affected timepoint as well as the preceding and following two timepoints

Statistical modeling & inference

Model type and settings	Mass-univariate linear models.
Effect(s) tested	We tested the main effect of four genetic variants and three psychiatric diagnoses on functional connectivity using general linear models.

Specify type of analysis: ☒ Whole brain ☐ ROI-based ☐ Both

Statistic type for inference (See Eklund et al. 2016)	Our analysis was conducted as a connectome wide analysis using mass univariate linear regression of each functional connection between 64 seed regions.
Correction	FDR

Models & analysis

n/a	Involved in the study
☐	☒ Functional and/or effective connectivity
☒	☐ Graph analysis
☒	☐ Multivariate modeling or predictive analysis

Functional and/or effective connectivity	Pearson correlation
